# Ghrelin Influences Novelty Seeking Behavior in Rodents and Men

**DOI:** 10.1371/journal.pone.0050409

**Published:** 2012-12-05

**Authors:** Caroline Hansson, Rozita H. Shirazi, Jakob Näslund, Heike Vogel, Corinna Neuber, Göran Holm, Henrik Anckarsäter, Suzanne L. Dickson, Elias Eriksson, Karolina P. Skibicka

**Affiliations:** 1 Department of Physiology/Endocrinology, Institute of Neuroscience and Physiology, The Sahlgrenska Academy at the University of Gothenburg, Gothenburg, Sweden; 2 Department of Clinical and Molecular Medicine, Institute of Medicine, The Sahlgrenska Academy at the University of Gothenburg, Gothenburg, Sweden; 3 Department of Pharmacology, Institute of Neuroscience and Physiology, The Sahlgrenska Academy at the University of Gothenburg, Gothenburg, Sweden; 4 Department of Metabolism and Cardiovascular Disease, Institute of Medicine, The Sahlgrenska Academy at the University of Gothenburg, Gothenburg, Sweden; 5 Department of Psychiatry and Neurochemistry, Institute of Neuroscience and Physiology, The Sahlgrenska Academy at the University of Gothenburg, Gothenburg, Sweden; The Scripps Research Institute, United States of America

## Abstract

Recent discoveries indicate an important role for ghrelin in drug and alcohol reward and an ability of ghrelin to regulate mesolimbic dopamine activity. The role of dopamine in novelty seeking, and the association between this trait and drug and alcohol abuse, led us to hypothesize that ghrelin may influence novelty seeking behavior. To test this possibility we applied several complementary rodent models of novelty seeking behavior, i.e. inescapable novelty-induced locomotor activity (NILA), novelty-induced place preference and novel object exploration, in rats subjected to acute ghrelin receptor (growth hormone secretagogue receptor; GHSR) stimulation or blockade. Furthermore we assessed the possible association between polymorphisms in the genes encoding ghrelin and GHSR and novelty seeking behavior in humans. The rodent studies indicate an important role for ghrelin in a wide range of novelty seeking behaviors. Ghrelin-injected rats exhibited a higher preference for a novel environment and increased novel object exploration. Conversely, those with GHSR blockade drastically reduced their preference for a novel environment and displayed decreased NILA. Importantly, the mesolimbic ventral tegmental area selective GHSR blockade was sufficient to reduce the NILA response indicating that the mesolimbic GHSRs might play an important role in the observed novelty responses. Moreover, in untreated animals, a striking positive correlation between NILA and sucrose reward behavior was detected. Two *GHSR* single nucleotide polymorphisms (SNPs), rs2948694 and rs495225, were significantly associated with the personality trait novelty seeking, as assessed using the Temperament and Character Inventory (TCI), in human subjects. This study provides the first evidence for a role of ghrelin in novelty seeking behavior in animals and humans, and also points to an association between food reward and novelty seeking in rodents.

## Introduction

The personality trait novelty seeking describes the response of a person to novel stimuli or situations in terms of tendency to explore, prefer or react positively to the novelty [1]. Importantly, this trait reliably predicts the tendency to develop drug abuse. High novelty seekers are thus more likely to experience drugs (e.g. [2]) and to develop compulsive drug taking [3].

A wealth of data suggests that drug seeking and novelty seeking have common neurobiological substrates. While food and drug seeking may be controlled by a partially overlapping mechanism, the relationship between food reward/seeking and novelty seeking behavior is as yet unknown. Considering the rapid rise in obesity, recently suggested to be influenced by pathological food reward behavior, by some even termed “food addiction”, the possible relationship between food reward and novelty seeking however is worth exploring.

The human trait of novelty seeking may be reflected by novelty seeking phenotypes in rodents. Supportively, the same tight relationship is found between chemical drug reward and novelty seeking in preclinical rodent studies [4]. Variation in the mesolimbic dopamine system may underlie differential responses in such models. Ventral tegmental area (VTA) dopamine lesion and nucleus accumbens (NAc) dopamine depletion thus reduce novelty seeking as measured by locomotor activity in response to novelty [5]. Moreover, rats displaying a relatively high locomotor response to novelty are characterized by enhanced dopamine activity in the NAc [3], [6]–[9], their dopamine neurons respond with more dopamine release to cocaine [10], [11], and their accumbal dopamine receptors are more sensitive to dopamine [12].

Ghrelin, a hormone produced in the stomach before meals and during states of hunger, has recently been associated with both drug and food reward behavior. While administration of ghrelin increases food [13], [14], alcohol [15] and cocaine [16], [17] reward behavior, pharmacological or genetic impairment of ghrelin neurotransmission reduces these behaviors. Moreover, ghrelin is tightly connected to the central dopamine system and central opioids. Ghrelin administration increases mesolimbic dopamine levels and changes expression of striatal dopamine receptors [18]–[20]. Ghrelin’s effects on food reward are also mediated, at least in part, by the central opioid system [21]. These previous findings provide strong though indirect support for a possible role for ghrelin in novelty seeking behavior.

Here we assessed the role of ghrelin in novelty seeking behavior in three complementary animal models of novelty seeking: 1) inescapable novelty-induced locomotor activity (NILA), 2) novelty place preference and 3) the novel object exploration. During the NILA test, rats are forced into a novel environment and their locomotor activity in response to this new environment is measured. This test is likely linked to stress responses as rats displaying a high NILA have high circulating levels of corticosterone [4]. In contrast, during a novelty preference test, rats can choose to explore a novel environment, the outcome of which is not linked to elevations in corticosterone [22]. Thus the two novelty models seem to be testing complementary and partially non-overlapping aspects of novelty seeking. Furthermore, while the inescapable NILA test is suggested to predict drug self administration in spite of negative consequences, novelty place preference may predict the propensity to develop compulsive drug taking after initial exposure [23], a high predicting development of addiction-like behavior. The novel object recognition task is based on the natural tendency of rodents to spend more time interacting with a novel object [24] and informs on recognition memory as well as novelty exploration tendency. 4) We also investigated the relationship between novelty seeking and food reward behavior, using the sugar motivated progressive ratio operant conditioning model. 5) In order to investigate the potential neural substrate underlying the novelty responses to ghrelin or the GHSR antagonist we investigated the effect of GHSR stimulation or blockade selectively in the VTA on the NILA response. 6) In addition to rodent behavioral studies, we evaluated the association between ten single nucleotide polymorphisms (SNP) in the genes encoding ghrelin (*GHRL*) and the *GHSR* and the personality trait novelty seeking in a Caucasian population.

## Materials and Methods

### Animals

Adult male Sprague-Dawley rats (n = 195; body weight 150–175 g at the start of testing) were used in the study. Standard chow (Harlan Teklad; Norfolk, UK) and water were available *ad libitum* in home cages. The animal room was maintained on a 12/12 hour light/dark cycle (lights on at 7 am), at 20°C and 50% humidity. All behavioral tests were conducted between 09∶00 and 16∶00 h in a testing room under dim, white light. All procedures were approved by the local Ethics Committee for Animal Experiments: Göteborgs djurförsöksetiska nämnd (GDN); permit number 314-10, 336-09.

### Drugs

Ghrelin 0.33 mg/kg (Tocris, Bristol, UK) or the GHSR antagonist JMV2959 1.5 mg/kg (AEZS-123, AeternaZentaris GMBH, Frankfurt, Germany [25]) were applied intraperitoneally (IP) and delivered at 1 ml/kg in saline solution. For the VTA microinjections all drugs were infused in a 0.5 µl volume of artificial cerebrospinal fluid (acsf), unilaterally at the following doses: ghrelin 1 µg/0.5 µl, JMV2959 10 µg/0.5 µl. The selected doses of ghrelin and the GHSR antagonist were previously shown to specifically affect food reward behavior, without producing non-specific motor effects [13], [20].

### Operant Conditioning

Food-induced operant conditioning training and testing were conducted in rat conditioning chambers (Med-Associates, Georgia, VT, USA) as in a previous study [26]. Rats (n = 31) were trained to press a lever for a 45 mg sucrose reward. Training was conducted in four stages: rats were first trained on an fixed ratio 1 (FR1) schedule (single press on the active lever resulted in the delivery of one sucrose pellet), followed by FR3 and FR5 (3 and 5 presses per pellet, respectively), where a minimum of 100 presses on the active lever per session was required for the advancement to the next schedule, culminating with progressive ratio conditioning until stable responding was achieved. Responding was considered stable when the number of pellets earned per session did not differ more than 15% for three consecutive sessions. Operant response testing was performed after the responses stabilized. Following operant testing the same rats (n = 31) were tested for their NILA responses during a 30 min period (see below for NILA protocol) to correlate the food motivation to novelty seeking. A separate group of rats was used for the experiments below.

### NILA

In the inescapable NILA test, rats are placed in a novel environment and their locomotor activity is read out as an index of novelty seeking. After IP injection of vehicle (saline, n = 61), ghrelin (n = 18) or the GHSR antagonist JMV2959 (n = 15), the rats were placed in a novel chamber 0.7 m×0.7 m×0.35 m [27] equipped with a grid of photocells and their locomotor activity was analyzed over a period of 60 min. Following this novelty reactivity test, vehicle injected rats were divided into high and low responder groups (upper and lower third of rats based on their total locomotor activity during a 60 min period [28]) and were further tested in the novelty preference and novel object exploration studies described below. Each test was separated by one week.

### Plasma Ghrelin Levels

In order to determine ghrelin (total and active) levels in high and low NILA responder rats, 40 rats were exposed to a NILA test for 30 min and their tail blood taken immediately after the NILA test. NILA test results were analyzed and upper and lower thirds of the rats based on their NILA activity were selected (n = 12 in each group). Blood samples from those selected rats were immediately centrifuged after the addition of AEBSF and EDTA. Plasma was collected and acidified with 1 M HCl. For quantification of total ghrelin levels a commercially available ELISA for rat/mouse (EZRGRT-91K; Millipore, Billerica, MA) was used and active ghrelin was measured using a rat/mouse ghrelin ELISA (EZRGRA-90K; Millipore; reported 100% specificity for active ghrelin and 0% for rat/mouse “des-octanoyl” ghrelin).

### Novelty Place Preference

The rats were exposed for 3 days, 30 min each day, to only one of two sides of a rectangular chamber (one side with black and white stripes, the other wooden with a marble PVC floor; sides counterbalanced across treatment). On the 3rd day rats received an IP injection of saline, ghrelin or JMV2959 10 min before the placement in the box with free access to both sides (familiar and novel). The test (duration of time in boxes) lasted 10 min. The rats were initially placed on the familiar side. Animals were considered to have entered a compartment when the two front paws and the head crossed the border line. The 10 min test was recorded with a video camera mounted above the boxes and later analyzed by an observer blinded to the treatment with the amount of time rats spent exploring the familiar and novel side being the parameter of interest.

### Novel Object Exploration

The apparatus was a wooden, square chamber (traditional open field; 1×1×0.5 m), divided into four zones. Black lines drawn on the floor divided each zone into four virtual 25 cm^2^ quadrants. Two types of objects to be discriminated were made of a neutral (e.g. odorless) material and were of different shapes: the sample-object, a ceramic cup 6 × 8 cm, and the novel object, a plastic water bottle (4.5 × 12 cm). A pilot study indicated that the amount of time spent interacting with both objects at baseline was similar, as previously published [29]. A video camera was mounted above the box to record the exploratory behavior during testing. Light intensity at the floor level was 40 lux.

On the testing day, each rat was brought to the testing room in their home cage and placed in the empty chamber for a 30 min habituation period. Immediately following this habituation, two identical copies of the sample object were placed in the chamber in adjacent quadrants with the animal being placed into an empty quadrant, facing away from the objects (sample session). The sample session was followed by a delay period (10 min) and then by the choice session. The short delay period was set to bias the test towards indicating novelty exploration by minimizing the influence of variation in memory formation processes. Drug injection (IP saline, ghrelin or GHSR antagonist) took place immediately after the sample session. During the choice session, a copy of the “sample” object remained and the other object was replaced by a new (i.e. novel) object. The object exploration during each test session was scored for a period of 60 sec. Object positions were counterbalanced between rats to avoid location bias. The apparatus was thoroughly cleaned between trials with 70% alcohol.

Time spent interacting/exploring the novel and familiar objects in sample and choice sessions was determined by an observer blinded to treatment status. “Exploration of an object” was defined as directing the nose to the object at a distance of less than 2 cm and/or touching it with the nose. Novel-object discrimination was determined by directly comparing the time spent investigating each object (walking past the object, backing into an object and tail-only contact being excluded). Any animal that did not exhibit a minimum of 4 sec of total contact with each of the objects in the sample session, and at least 1 sec contact with either object in choice session, was excluded from the study. One animal was removed from each treatment group based on this criterion.

### VTA Directed Ghrelin or GHSR Antagonist and NILA

Surgery: Rats (n = 24) were implanted with a guide cannula targeting the VTA (26 gauge; Plastics One, Roanoke, VA, USA). The guide cannula was placed 2 mm above the target site, and an injector extending 2 mm from guide cannula was used for injections. To target the VTA our previously confirmed coordinates were chosen [13]: ±0.75 mm from the midline, 5.7 mm posterior to bregma, and 6.6 mm ventral to dura mater, with injector aimed 8.6 mm ventral to the dura. The cannula was attached to the skull with dental acrylic and jeweler’s screws and closed with an obturator, as described previously [26]. Injection was verified post mortem by injection of India ink at the same volume (0.5 µl) as was used throughout the study. Only subjects with the correct placement were included in the data analysis.

Behavioral testing: NILA test was conducted as described above 20 min after VTA directed microinjections of the GHSR ligands or vehicle.

### Statistics

All parameters obtained from the rodent studies were analyzed by analysis of variance (ANOVA) followed by Bonferroni’s multiple comparison test as appropriate when more than 2 groups were compared or t-test for two group result analyses. For correlation analysis Pearsons r was calculated. All statistical analyses were conducted using Graph Pad Prism software (La Jolla, CA, USA).

### Ghrelin Receptor SNP Association with Novelty Seeking

#### Subjects

The studied subjects, 125 men born in 1944 and 192 women born in 1956 and living in Gothenburg, Sweden, were drafted from a larger cohort recruited from the general population as previously described [30]–[33]. Only subjects that completed the personality trait assessment and were Caucasian were included in the present study. The study was approved by the Regional Research Ethics committee at the University of Gothenburg. All participants provided written informed consent.

#### Genotyping

All individuals were genotyped for 6 tag SNPs in *GHRL* (the gene encoding ghrelin) and 4 tag SNPs in *GHSR* (the gene encoding the receptor for ghrelin). The selected SNPs were previously identified and selected to cover genetic variation in the selected genes [34].

Venous blood was collected from each subject, and genomic DNA was isolated using the QIAamp DNA blood Mini Kit (Qiagen, Chatsworth, CA, USA). Genotyping was performed at Sequenom Inc. in Hamburg, Germany, using the Sequenom iPLEX® Gold assay and MassARRAY® MALDI-TOF mass spectrometry platform in accordance with the manufacturer’s instructions (Sequenom Inc., San Diego, CA, USA). Primers for PCR amplification and sequencing were designed using the Sequenom MassARRAY® System Designer software.

#### Assessment of personality traits

Novelty seeking temperament was assessed using the Swedish translation of the Temperament and Character Inventory (TCI) scale. The TCI is a self-administered true-false questionnaire that measures temperament and character along seven personality dimensions, of which four are claimed to measure temperament (novelty seeking, harm avoidance, reward dependence and persistence) and three to measure character (self-directedness, cooperativeness and self-transcendence) [35]. The TCI test scores were standardized using normative data (T scores) to have an expected mean of 50 and standard deviation of 10.

#### Statistical analysis

Hardy Weinberg equilibrium was tested using Haploview. Association between the polymorphisms and the personality trait novelty seeking was analyzed using linear regression. P-values were corrected for multiple testing using permutation tests due to the linkage disequilibrium between the SNPs.

## Results

### Food Reward and NILA

**Figure 1:**
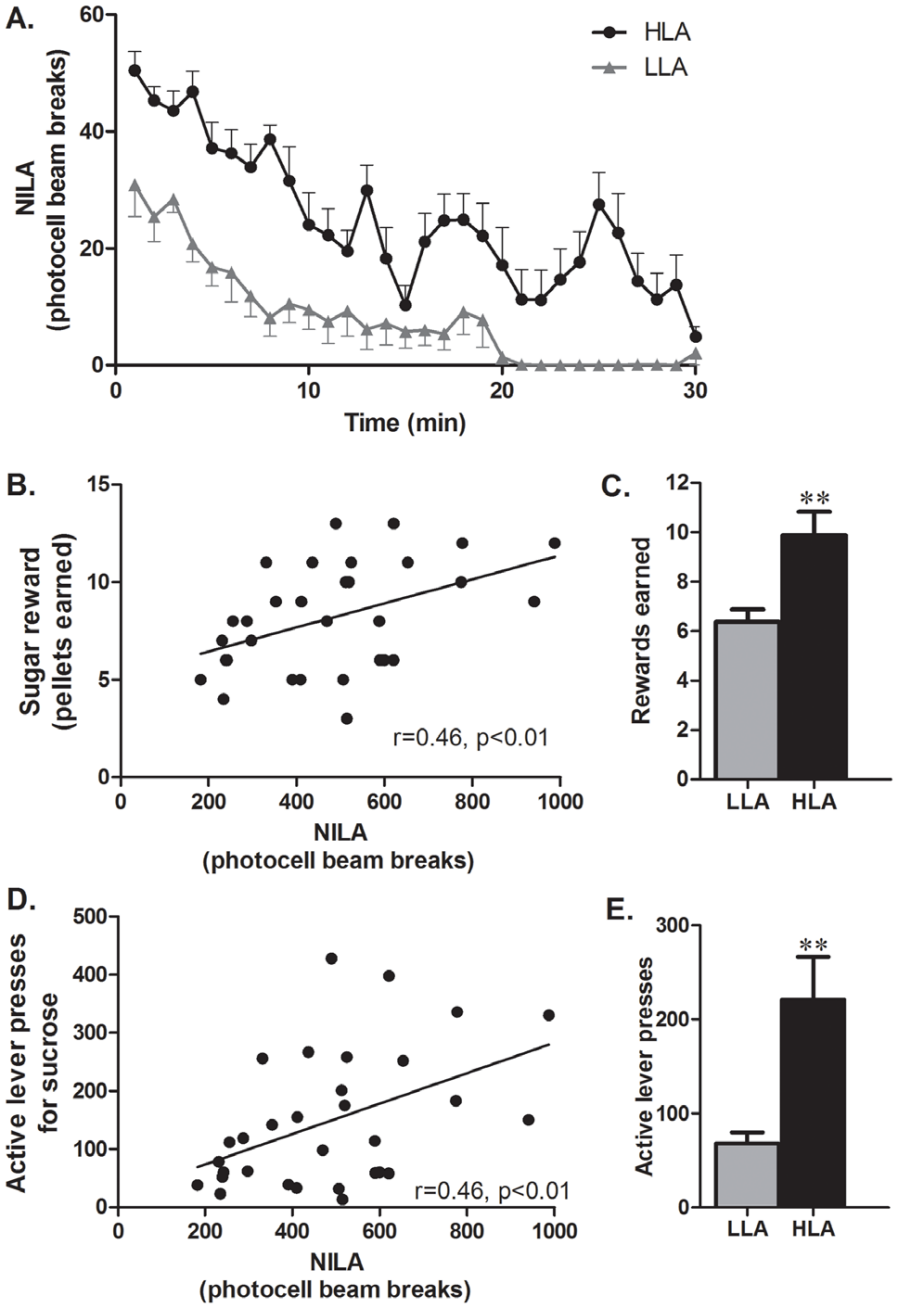
Food reward seeking and NILA are behaviorally related traits. A. Outbred Sprague-Dawley rats were divided into high NILA (HLA, n = 8) and low NILA rats (LLA, n = 8); the NILA response of LLA rats was significantly lower compared to that of HLA rats during the 30 minute period of testing. B. Number of sugar rewards earned on a progressive ratio schedule was significantly correlated with the NILA response (n = 31). C. High NILA rats earned more sugar rewards than low NILA rats. D. NILA response was significantly correlated with the amount of work rats were willing to expand for sugar rewards. E. High NILA rats display greater lever pressing rates for sugar rewards. **P<0.005.

Food reward behavior as measured by the number of sucrose pellets earned (Figure 1B), or by the number of active lever presses for sucrose (Figure 1D), was significantly correlated with NILA (n = 31; r = 0.46, p<0.01 and r = 0.46, p<0.01 for sugar rewards earned and active lever presses respectively). When rats were selected for high versus low NILA responses (top or bottom n = 8 for each group, Figure 1A), a striking difference in their respective food reward behavior emerged, where high NILA rats earned nearly 50% more sugar rewards (Figure 1C) and pressed the lever for sucrose nearly 3 times more (Figure 1E).

### Effect of Peripheral Ghrelin on NILA

Ghrelin did not alter cumulative novelty reactivity at 60 min (549±16 vs. 481±35 cumulative locomotor activity counts; ns, vehicle n = 61 vs. ghrelin n = 18 respectively) and only briefly increased activity during the first minute of exposure to the new environment (56±2 vs. 63±3; p<0.05 vehicle n = 61 vs. ghrelin n = 18 respectively). However blockade of GHSR, significantly decreased novelty reactivity, starting at the second minute of testing until the end of the test at 60 min (549±16 vs. 446±49 cumulative locomotor activity counts; p<0.05, vehicle n = 61 vs. JMV2959 n = 15 respectively).

### Plasma Ghrelin Levels

**Figure 2:**
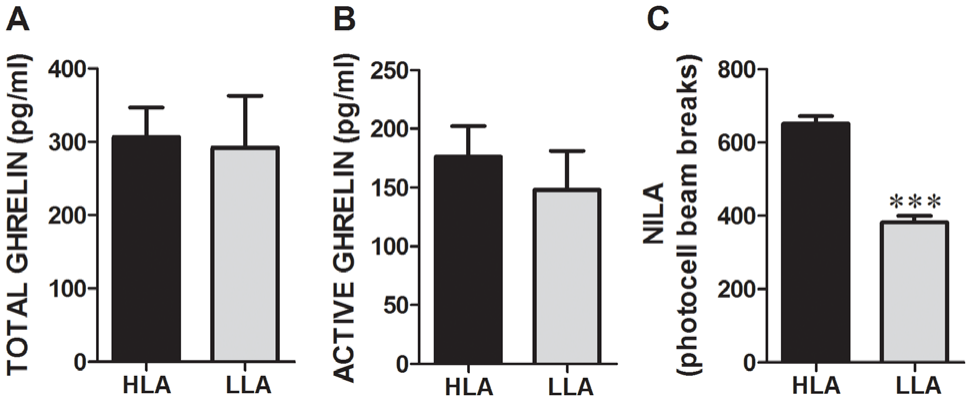
Plasma ghrelin levels in high and low NILA rats. A. Total ghrelin levels and B. active ghrelin levels are not different in animals with low (n = 12) vs. high (n = 12) NILA. C. The same rats display a markedly different activity level in the novel environment. Data on the bar graphs represent mean ± SEM. ***P<0.0005.

No significant differences in both total and active plasma ghrelin levels after 30 min of the NILA test have been detected for the low vs. high NILA rats despite prominent differences in their NILA response levels (p<0.0001, students t test; Figure 2). No significant correlation of the NILA and active (Pearson r = 0.24, p = 0.29) or total (Pearson r = 0.13, p = 0.6) ghrelin levels was detected (data not shown).

### Effect of Ghrelin on Novelty Place Preference

**Figure 3:**
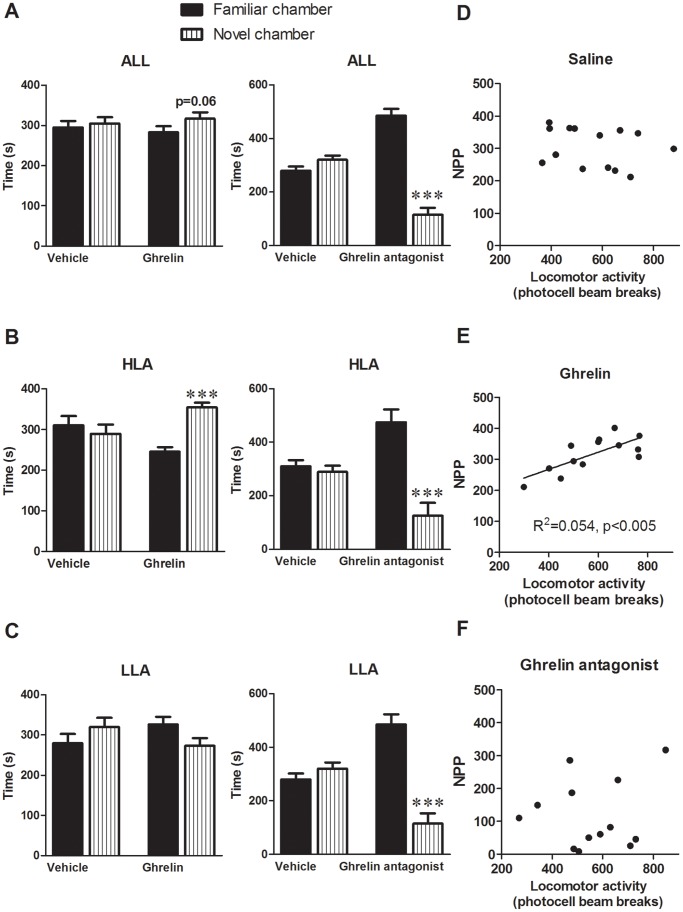
Role of ghrelin in novelty place preference. A. Ghrelin-treated rats tend to have slightly higher preference for exploring a novel environment. In contrast those that received GHSR antagonist display much lower preference for a novel environment as compared to the more familiar one. B. Ghrelin markedly increases and the GHSR antagonist strikingly decreases exploration of a novel environment in high NILA rats (HLA). C. When only the low NILA (LLA) rats are considered ghrelin does not significantly alter the place preference, however GHSR antagonist is still effective at reducing the preference. ***P<0.0005. Ghrelin alters the relationship between preference for novelty and NILA. D. Novelty place preference (NPP, here time spent exploring the novel environment) is not correlated with locomotor activity during NILA at baseline. E. The two traits become significantly correlated after ghrelin treatment. F. GHSR antagonist does not influence the correlation.

As mentioned above, the rats used for the novelty preference test were the 1/3 of the animals being most (“high NILA”) (693±18 locomotor activity counts, n = 20) or least (“low NILA”) (493±17, n = 21) active in the NILA test, respectively. The effect of GHSR stimulation or blockade on preference for the novel environment was examined in both high and low inescapable activity rats (Figure 3). During the preference test, ghrelin-injected rats, but not vehicle-injected rats, showed a slight preference for the novel compartment (p = 0.06). Interestingly, this ghrelin-induced preference was driven solely by the rats that scored high on the NILA test showing a large preference for the novel chamber when injected with ghrelin (p<0.001) but not under vehicle condition. Low novelty reactivity rats showed no preference for the novel environment after either ghrelin or vehicle administration. The interaction between the high and low NILA rats and ghrelin’s effect on novelty preference was further confirmed by a 2-way ANOVA analysis (drug×NILA group) of time spent in the novel environment (F _(1,23)_ = 8.05, p = 0.009). The GHSR antagonist, however, uniformly reduced the preference for the novel chamber for both high (p<0.001) and low scoring rats (p<0.001).

Here we confirm previous literature findings indicating that place preference and NILA are not correlated (Figure 3D), but also extend them to show that the effect of ghrelin on place preference is significantly correlated with baseline reactivity/NILA (Figure 3E). No correlation was found for the GHSR antagonist (Figure 3F). Thus, while at baseline preference and reactivity are not associated, ghrelin administration brings out the connection between those two novelty seeking measures. What follows is that high NILA ghrelin-injected rats show a significantly higher amount of time spent on the novel side as compared to low reactivity ghrelin-injected rats (p<0.005).

### Effect of Ghrelin on Novel Object Exploration

**Figure 4:**
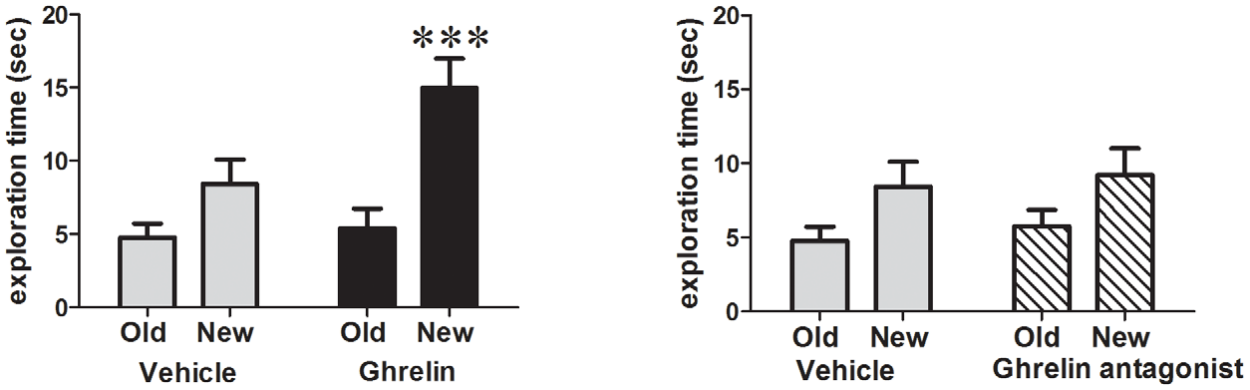
Ghrelin induces a significant preference for exploration of a novel object in rats that do not show a preference for a novel object at baseline. GHSR blockade does not alter this behavior. ***P<0.0005.

Ghrelin significantly increased exploration of the novel object in both high and low reactivity rats, while the vehicle-injected rats did not preferentially explore the novel object (one way ANOVA for all subjects F_(3,48)_ = 11.9, p<0.0001, high NILA only: F_(3,18)_ = 4.5, p<0.05, low NILA only: F_(3,20)_ = 10.7, p<0.0001). Post hoc test results indicate that the time spent exploring the novel object is significantly higher in the ghrelin-treated groups only. Two-way ANOVA analysis did not indicate a significant interaction between the time spent exploring the novel object and the NILA group (F_(1,22)_ = 2.8, p = 0.11). GHSR antagonist injected rats, like those injected with vehicle, did not show a significant preference for exploring the new object (Figure 4).

### Effect of VTA Ghrelin on NILA

**Figure 5:**
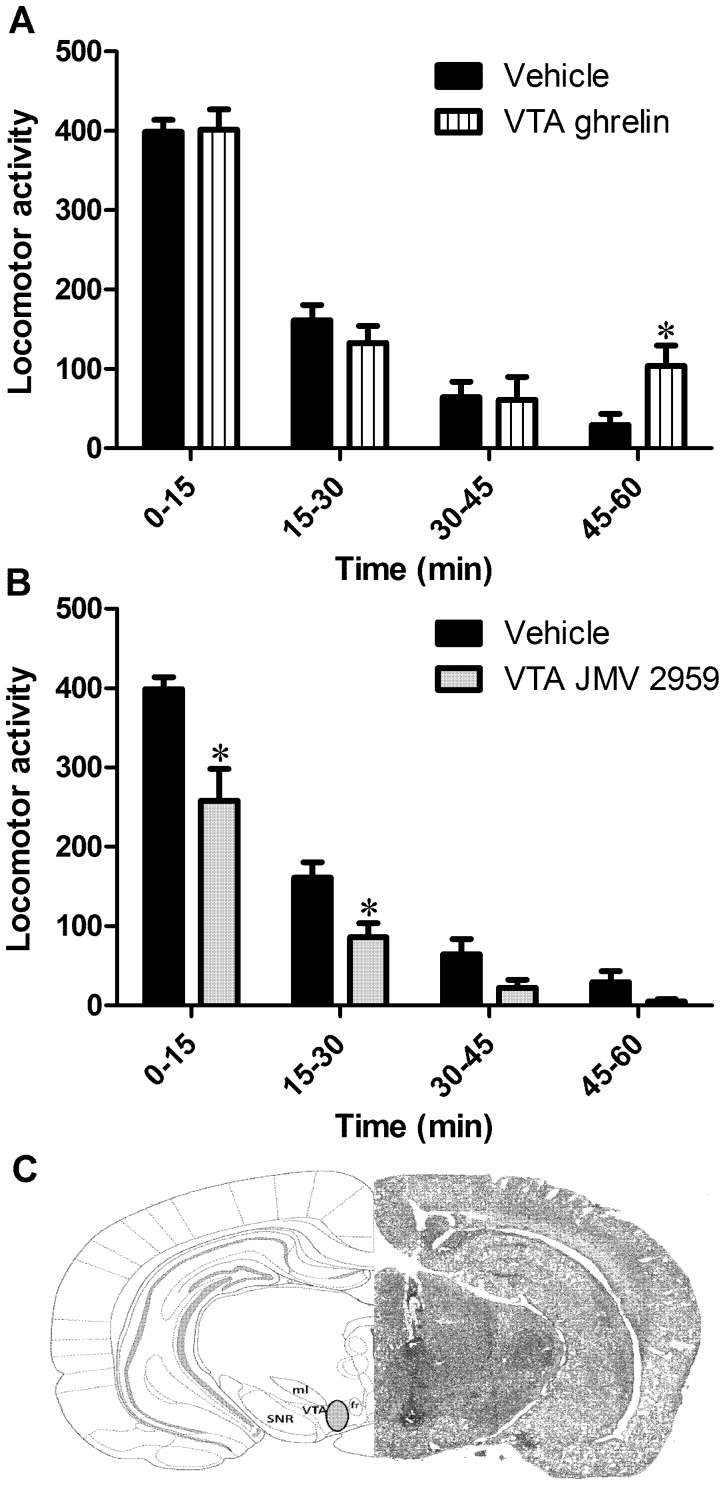
Role of the VTA ghrelin and GHSR in the NILA response. A. Rats that received the VTA directed ghrelin microinjection had an elevated NILA response after 45 min of the NILA test. B. Conversely rats that received a VTA microinjection of the GHSR antagonist JMV2959 displayed a reduced NILA response. C. Rat brain section (right) and equivalent panel from the rat brain atlas (left) showing an example of the VTA microinjection used. *P<0.05. SNR, substantia nigra, reticular part; ml, medial longitudinal fasciculus; fr, fasciculus retroflexus; VTA, ventral tegmental area.

VTA microinjected ghrelin did not alter novelty reactivity at the 0–45 min time point. It did, however, increase novelty reactivity significantly after 45 min until 60 min (which was the end of the testing period). The rats that received ghrelin displayed nearly a threefold elevation in their activity compared to the vehicle-injected rats at this time point (Figure 5A; p<0.05). In order to confirm that the dose of ghrelin was physiologically active, 30 min food intake was measured immediately after the VTA injection of ghrelin or vehicle. We found that vehicle injected rats did not consume any chow (as expected for rats in the mid light cycle) whereas those injected with ghrelin increased their consumption of chow during the 30 min measurement (mean intake 0.1±0.05 g and 1.8±0.6 g for vehicle and ghrelin respectively, p<0.05). This orexigenic effect of VTA microinjected ghrelin is consistent with several previous reports [13], [21], [36]. VTA selective blockade of GHSR, potently and significantly decreased novelty reactivity, for the first 30 min of the test (Figure 5B; p<0.05).

### Association between Novelty Seeking and GHSR SNPs

**Figure 6:**
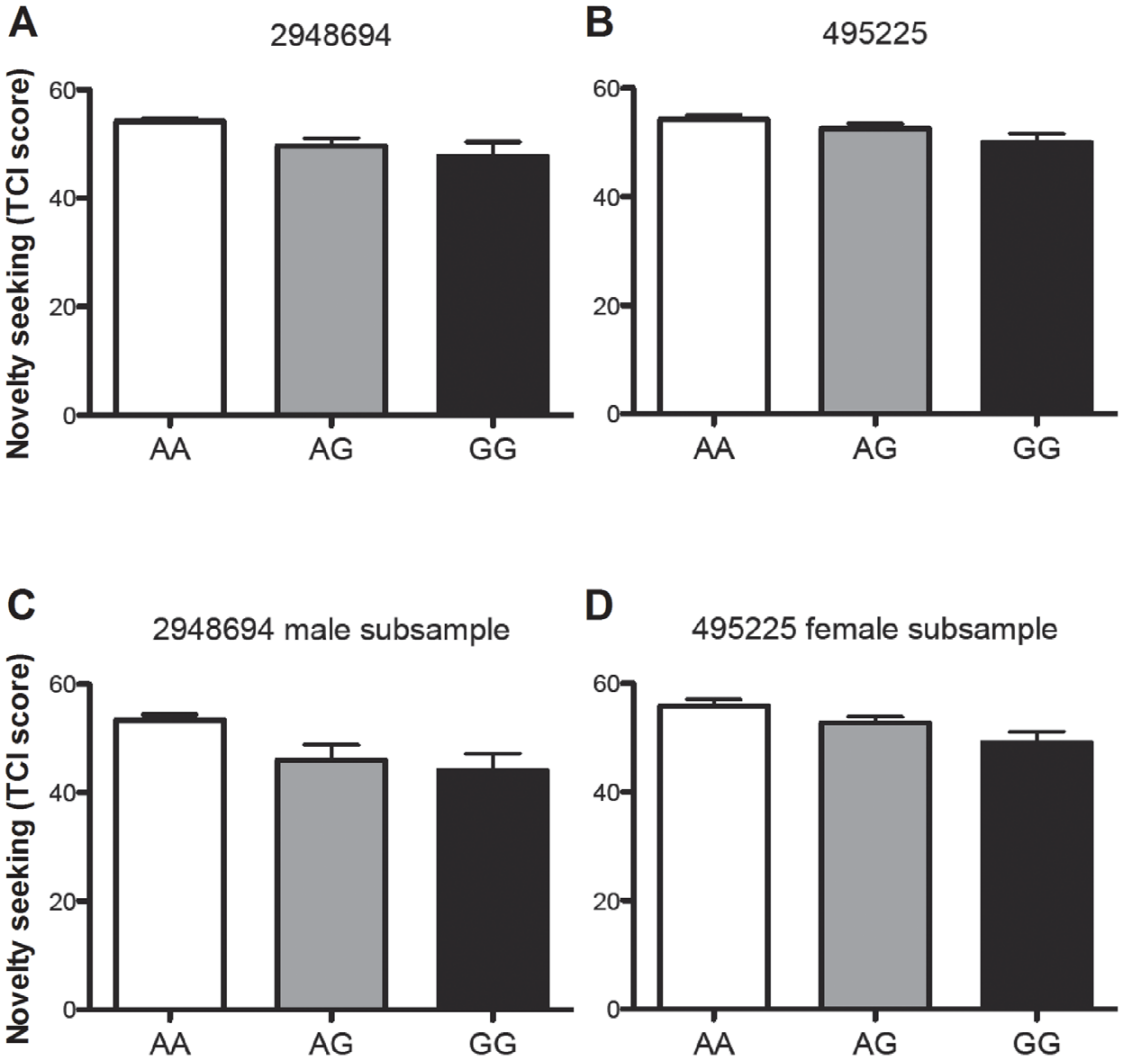
The *GHSR* SNPs rs2948694 and rs495225 are significantly associated with novelty seeking (p = 0.002 and p = 0.026, respectively). For both rs2948694 and rs495225, the less common G/G genotype is associated with lower scores on the personality trait novelty seeking.

The genotype frequencies were in Hardy-Weinberg equilibrium. The *GHSR* SNPs rs2948694 (intron 1) and rs495225 (exon 1, silent) were significantly associated with novelty seeking (p = 0.002 and p = 0.026 respectively). Further analyses of the male and female subgroups revealed that rs2948694 had a stronger association with novelty seeking in the male subsample (p = 0.003), while rs495225 had a more prominent association with novelty seeking in the female subsample (p = 0.004), although there were no significant differences between the male and the female subgroups for neither of these SNPs. For both rs2948694 and rs495225, the less common G/G genotype was associated with lower scores on the personality trait novelty seeking (Figure 6). The tag SNPs analyzed, the genotype frequencies, the ß-values, the p-values for the association tests using linear regression and the corrected p-values using permutation test are listed in Table 1.

**Table 1:** The analyzed tag SNPs, the genotype frequencies and the p-values for the association tests using linear regression.

*GHRL*						
rs26802	TT	GT	GG	β	P	P_corrected_
Total sample	165	126	23	−1,15	0,230	0,897
Male subsample	64	51	9	1,29	0,405	
Female subsample	101	75	14	−**2,71**	0,026*	
**rs4684677**	TT	AT	AA	β	P	P_corrected_
Total sample	267	39	1	−1,97	0,259	0,926
Male subsample	110	13	0	−5,97	0,053	
Female subsample	157	26	1	−0,42	0,842	
**rs42451**	CC	CT	TT	β	P	P_corrected_
Total sample	185	110	15	0,02	0,988	1,000
Male subsample	72	44	6	2,59	0,119	
Female subsample	113	66	9	−1,65	0,217	
**rs35680**	TT	CT	CC	β	P	P_corrected_
Total sample	82	146	72	−0,52	0,545	1,000
Male subsample	29	62	28	−1,86	0,191	
Female subsample	53	84	44	0,31	0,772	
**rs34911341**	CC	CT	TT	β	P	P_corrected_
Total sample	314	3	0	8,30	0,180	0,829
Male subsample	124	1	0	7,93	0,463	
Female subsample	190	2	0	8,33	0,270	
**rs 696217**	GG	GT	TT	β	P	P_corrected_
Total sample	254	58	4	1,42	0,300	0,955
Male subsample	97	26	1	0,25	0,908	
Female subsample	157	32	3	2,28	0,194	
***GHSR***						
**rs2948694**	**AA**	**AG**	**GG**	**β**	**P**	**P_corrected_**
Total sample	251	58	6	−**4,11**	0,002*	0,015*
Male subsample	105	17	3	−**6,22**	0,003*	
Female subsample	146	41	3	−3,07	0,062	
**rs572169**	CC	CT	TT	β	P	P_corrected_
Total sample	140	143	32	−0,02	0,979	1,000
Male subsample	48	61	16	0,56	0,697	
Female subsample	92	82	16	−0,22	0,855	
**rs2232165**	GG	AG	AA	β	P	P_corrected_
Total sample	289	21	1	−2,73	0,219	0,887
Male subsample	114	7	1	0,60	0,860	
Female subsample	175	14	0	−5,38	0,068	
**rs495225**	AA	AG	GG	β	P	P_corrected_
Total sample	151	129	32	−**2,00**	0,026*	0,215
Male subsample	65	42	13	−0,24	0,862	
Female subsample	86	87	19	−**3,34**	0,004*	

*GHRL, pro-ghrelin gene; GHSR, growth hormone secretagogue receptor gene*; β, β-value describing the slope of the curve in the linear regression model; p, p-value using linear regression; p_corrected_, p-value corrected for multiple testing using permutation test.

## Discussion

The stomach produced orexigenic peptide ghrelin has long been associated with its actions on the homeostatic brain systems. Only recently it became apparent that it plays a crucial role in the mesolimbic system and enhances drug, alcohol and food reward. The current results extend these findings to include effects of ghrelin onto a behavior closely associated with drug reward-novelty seeking. We found that central ghrelin signaling contributes to novelty seeking in three complementary models in rodents. While, ghrelin elevated preference for a novel environment as well as preferential exploration of a novel object, GHSR blockade reduced preference for a novel environment and also inescapable novel environment exploration. These preclinical data were complemented by results of the genetic association study in which two SNPs in the GHSR were associated with the novelty seeking trait in human subjects. Collectively, our data suggest an important role for ghrelin in the modulation of novelty seeking behavior.

While there is an abundance of data linking drug self-administration or reward with novelty seeking, little is known about the possible relationship of novelty seeking and food reward. Both reward processes can be localized to the mesolimbic circuitry with a prominent role for dopamine. Here we show that sugar reward behavior is positively correlated with NILA, as rats exhibiting higher activity in a novel environment are willing to work much harder for a sugar reward. These results are consistent with one previous report indicating that preference for a novel environment and object may be linked to sucrose self-administration [37].

Thus neurochemical and behavioral cross-talk exists between food reward, drug addiction and ghrelin, and all three are linked to novelty seeking, suggesting common mechanisms. Collectively, this suggests a complex web of interactions of these three behavioral traits under the primary control of the mesolimbic dopamine system, and with gut-produced ghrelin exerting a modulatory role.

Several neurobiological substrates common to ghrelin and novelty seeking could underlie the action of ghrelin on novelty seeking. Our data showing that VTA selective ghrelin microinjection increases, and importantly, VTA GHSR antagonist microinjection decreases, novelty reactivity, suggest a crucial role of the VTA in ghrelin’s effects on novelty seeking. Dopamine increases all novelty responses and is itself elevated by ghrelin, making it a likely mediator of the effect of ghrelin on novelty [18]–[20]. However, ghrelin in the VTA can also affect dopamine indirectly via other mediators, e.g. GABA and opioids, that are also linked to novelty seeking [38]. For example GHSRs are located on GABAergic neurons in the VTA that regulate VTA dopamine neuron activity, and GABAergic signaling within the VTA has been implicated in novelty seeking [18]. Signaling via opioid receptors is required for ghrelin-induced increase in food-reward behavior and elevated opioids have been linked to novelty behavior [21]. Thus a complex web of neurochemical changes, ultimately leading to elevated mesolimbic activity and increased novelty seeking, might be involved.

While the enhancing effects of ghrelin administration on novelty seeking were clear and potent in the novelty place preference test and the novel object exploration test, no effect of peripherally applied ghrelin on the NILA test was detected. We may infer that signaling at GHSR is necessary but not sufficient to induce NILA alone. Inescapable novelty and novelty choice may be controlled by common as well as partly divergent circuitry. Novelty preference and NILA have previously been shown to be two independent, uncorrelated, measures of novelty seeking [23]. Here we confirm these previous findings, but also extend them to show that the effect of ghrelin on preference is significantly correlated with baseline NILA. Thus it is also possible that the effects of ghrelin on NILA require a high baseline NILA but unfortunately, due to our methodological constraints, we were not able to determine this relationship. It is also possible that ghrelin primarily affects brain circuits controlling novelty choice. In line with this hypothesis, a well-known central mediator of the effect of ghrelin on food intake and motivation, NPY and its Y1 receptor may have opposing effects on inescapable novelty and novelty choice. NPY Y1 stimulation thus reduced activity in an inescapable environment whereas it seemed to contribute to increased novelty seeking in a free choice novelty as well as novel object exploration test [39], [40]. Alternatively peripheral ghrelin injection could allow access to both stimulatory (in the VTA through dopamine) and inhibitory (via NPY in the hypothalamus) pathways producing a net result of no significant effect on the NILA. This might be supported by our data showing a stimulatory effect of ghrelin on NILA when ghrelin stimulation is limited to the VTA.

Interestingly GHSR antagonists might affect dopamine signaling irrespective of the presence of ghrelin [41], as ghrelin receptors have been shown to dimerize with several dopamine receptors and alter the dopamine evoked activation [41]. Thus it is possible for the GHSR antagonist to reduce novelty reactivity via a ghrelin-free GHSR receptor and consequently disrupt dopamine signaling required for NILA.

In the novelty place preference test, ghrelin induced a divergent effect on the preference for the novel environment in high vs. low NILA rats. While ghrelin was clearly effective in high NILA rats, it did not increase the preference for novelty in the low NILA rats. As already discussed, there is a wealth of reported behavioral and neurochemical differences between high and low NILA rats, and it is possible that one or many of these differences contribute to the differential effects of ghrelin obtained here. A more responsive dopamine system could have facilitated the modulatory action of ghrelin. A differential behavioral response in these two groups has been reported previously: for example, inactivation of the central nucleus of the amygdala is effective only in high, but not low NILA rats, at reducing amphetamine self-administration [42]. Thus, in the case of the novelty place preference, ghrelin appears to select out the vulnerable (high NILA) population.

Both chronic [43], [44] and acute stress [45] can elevate circulating ghrelin levels. Ghrelin can also elevate corticotropin-releasing hormone (CRH) neuron activity in the paraventricular nucleus (PVN) of the hypothalamus. These interactions of ghrelin with the stress axis connect well with its effects on novelty seeking since animals showing elevated novelty responses (here high NILA rats) also show enhanced and prolonged stress axis activation with elevated CRH mRNA in the hypothalamic PVN [46] and elevated plasma corticosterone levels [3]. Thus, the stress axis might be involved in ghrelin’s effects on novelty seeking. However, the fact that direct microinjection of ghrelin or the antagonist in the VTA was sufficient to change NILA behavior indicates that the involvement of hypothalamic PVN and the HPA axis might not be needed for ghrelin’s effects on novelty seeking. Also unlike circulating corticosterone, ghrelin levels were similar in high and low NILA rats during the novelty exposure. It seems unlikely, therefore, that differences in ghrelin secretion provide the main driver of the changes in the circulating corticosterone and also that circulating corticosterone elevation might not be sufficient in the novelty paradigm to elevate ghrelin levels.

Polymorphisms within the systems found to contribute to novelty seeking in rodents are often associated with novelty seeking in human subjects; this has, for example, been suggested for polymorphisms in dopamine-related genes [47]–[49]. For example, while the lack of dopamine D4 receptors in mice results in a lower novelty seeking behavior [50], polymorphisms in the dopamine D4 receptor gene, *DRD4*, have been associated with novelty seeking and problematic alcohol use in humans [51]. Likewise, while we in this study show ghrelin receptor activation to influence novelty seeking in animals, we were also able to identify associations between SNPs in the *GHSR* gene and the trait novelty seeking in humans. The less common genotype (G/G) of two *GHSR* SNPs, rs2948694 and rs495225, were thus associated with lower scores on the personality trait novelty seeking.

Given the association between novelty seeking and food reward, our observation is partly in line with previous studies suggesting an association of ghrelin or *GHSR* SNPs with obesity or overeating (see [52]). An association of rs2948694 with a high body weight has thus been reported previously in a Spanish population [34]; moreover, rs495225 was also found to be associated with body weight, though this finding was not replicated when an additional larger sample size was included [53].

Our results also highlight the need to consider the satiety state of the subject during novelty testing, as this could affect ghrelin levels and thus the novelty-seeking behavior. In both rodents and humans, ghrelin levels in blood display distinct diurnal patterns, which might be important to consider when analyzing novelty seeking-related behavior [54], [55].

There are indications that in the obese state, at least in rodents, ghrelin may no longer be effective at inducing an orexigenic response and increasing motivated behavior [56], [57]. This lack of effect of ghrelin in obesity might also be of importance for our suggested role for ghrelin in novelty behavior, especially considering that the neurocircuitry underlying the effects of ghrelin on motivated behavior and novelty partly overlap.

The results obtained here are consistent with an evolutionary perspective where the main role for ghrelin is to indicate the lack of nutrients in the stomach and initiate behaviors to counteract that. Thus, the reason for ghrelin to increase novelty seeking might be the advantage of locating new sources of food. Taken together, our data suggest that ghrelin influences a range of novelty seeking behaviors. Our data are strengthened by consistent results obtained with ghrelin across several novelty seeking tests. The two-step hypothesis [4] describes inescapable novelty responses as predictors of the propensity to experience drugs, and novelty preference as a predictor of the transition to addiction/compulsive intake. As judged by the current results, GHSR antagonist could affect both stages, offering an interesting therapeutic target for both drug and food reward disorders.
